# Policy Series: Building Momentum for Diversity, Equity, and Inclusion in Geriatrics and Gerontology Education

**DOI:** 10.1093/geroni/igab046.339

**Published:** 2021-12-17

**Authors:** Jennifer Severance, Barbara Gordon, Brian Lindberg

## Abstract

With an increasingly multicultural and diverse older adult population, health care professionals must be prepared to serve older adults from varied backgrounds and marginalized communities; address health determinants and disparities; and promote diversity, equity, inclusion, and empathy within systems of care. The National Association for Geriatrics Education (NAGE) is a non-profit organization representing geriatric and gerontology education and training programs, including Health Services and Resource Administration (HRSA) funded Geriatric Workforce Enhancement Programs (GWEPs), and Geriatric Academic Career Awardees (GACAs). The 44 GWEPs focus on improving health outcomes for older adults by enhancing geriatrics and primary care training of the healthcare workforce. The 26 GACA awards support leaders in Age-Friendly health care transformation and interprofessional clinical geriatrics training. This symposium examines the role both programs have in reducing racial health disparities in older adults by promoting increased diversity of the geriatrics/gerontology workforce and advancing public policies for racial equity and inclusion. First, presenters will introduce the NAGE Diversity and Racial Equity Workgroup that supports a broader and unified effort across GWEPs and GACAs for equity and inclusion in geriatrics and gerontology education. Presenters will then share strategies to mobilize system-level changes within their institutions. Finally, examples of progress showcase individual GWEP and GACA projects and partnerships aimed at reducing racial health disparities within a multidimensional and local context. Presenters discuss strategies and opportunities to disrupt and transform health professions education at multiple levels and implications for policies supporting optimal aging for all older adults.

